# Differences in Clinical, Epidemiological, and Pathological Features of Breast Cancer in the Saudi Population: An Analytical Cross-Sectional Single Institution Study

**DOI:** 10.3390/healthcare13070737

**Published:** 2025-03-26

**Authors:** Sajid Durrani, Saad Alamri, Sojib Bin Zaman, Yosef Alobaisi, Abdullah Bany Hamdan, Musa Alharbi, Jude Howaidi, Khalil Alamri, Filwah Almarzouq, Alaa Alyahyawi

**Affiliations:** 1 Comprehensive Cancer Center, King Fahad Medical City, Riyadh 11525, Saudi Arabia; sdurrani@kfmc.med.sa (S.D.); ahamdan@kfmc.med.sa (A.B.H.); musaalharbi@gmail.com (M.A.); 2 Research Center, King Fahad Medical City, Riyadh 11525, Saudi Arabia; salamri@kfmc.med.sa; 3 Department of Health Sciences, James Madison University, Harrisonburg, VA 22807, USA; zamansb@jmu.edu; 4 Pathology and Clinical Laboratory Medicine Administration, King Fahad Medical City, Riyadh 11525, Saudi Arabia; yalobaisi@kfmc.med.sa; 5 Clinical Pharmacy Department, King Fahad Medical City, Riyadh 11525, Saudi Arabia; jhewaidi@kfmc.med.sa (J.H.); khalil_alamri@outlook.com (K.A.); falmarzouq@kfmc.med.sa (F.A.)

**Keywords:** Ki67 index, biomarker, molecular subtype, invasive ductal carcinoma, Saudi Arabia

## Abstract

**Background:** In Saudi Arabia, breast cancer is the most common malignancy among women, significantly impacting cancer-related morbidity and mortality. The country’s unique demographics and rapid socioeconomic development contribute to distinct breast cancer patterns. **Objective:** To analyze demographic and pathological characteristics of breast cancer with an emphasis on associations between the Ki67 proliferation index, tumor stages, and molecular subtypes. **Materials and Methods:** An analytical cross-sectional study was conducted on 294 breast cancer patients from 2013 to 2019, recruited from the Comprehensive Cancer Center at King Fahad Medical City, Riyadh, Saudi Arabia. A one-way ANOVA and logistic regression were used to identify risk factors associated with elevated Ki67 levels. Significance was set at a 95% confidence level. **Results:** The mean age of patients was 51.58. Among them, 67% were overweight/obese, 21.1% were diabetic, and 17% were hypertensive. Approximately 28.9% of all tumors were classified as T3, 46.9% as Grade III, and 40% as Stage IV. Invasive ductal carcinomas (83.9%) were the most common. ER, PR, and HER2 expression were positive in 63.4%, 54.3%, and 34.9% of patients, respectively, with a high Ki67 index in 50.7%. As compared to Grade I cancer, grade II cancer increased the likelihood of elevated Ki67 by 41 times (*p* = 0.001), Grade III cancer by 7.43 times (*p* = 0.001), and Stage IV cancer by 2.26 times (*p* = 0.03). Conversely, invasive lobular carcinomas and other cancer types were significantly less likely to have high Ki67 levels (*p* < 0.05). **Conclusions:** Elevated Ki67 appeared to predict higher tumor grades and certain molecular subtypes cancer.

## 1. Introduction

Breast cancer poses a major global health challenge as it is the most frequently diagnosed cancer worldwide [1,2], with approximately 2.26 million cases reported in 2020, and it remains the leading cause of cancer-related deaths among women [3]. Despite advancements in breast cancer care, significant disparities persist in screening, diagnosis, and management, especially in rural and semi-urban areas where access to healthcare services remains limited [4]. In developing countries and under-resourced settings, the escalating burden of the disease has crucial implications for preventive and therapeutic policies [5].

The American Cancer Society anticipates that 316,950 women and 2800 men will be diagnosed with invasive breast cancer by 2025 [6]. Detecting breast cancer early and implementing effective treatment methods has significantly improved survival rates, with a 5-year relative survival rate of 99% if caught at an early stage [6]. However, the burden of breast cancer is particularly exacerbated in developing countries and among rural and underserved populations due to limited health infrastructure and resources [7]. An increase in mortality rates has accompanied a significant rise in breast cancer incidence [8].

In countries with low-middle sociodemographic indices, breast cancer incidence increased significantly from 1990 to 2019 in comparison to countries with a middle sociodemographic index [8]. As a result, improved preventive and therapeutic policies are urgently needed to combat the growing burden of breast cancer in these regions [7]. Increasing access to screenings and diagnostics, improving healthcare infrastructure in rural and underserved areas, and implementing targeted public health interventions are all necessary to address the disparities in breast cancer care [9]. In these areas, action can be taken to reduce the global burden of breast cancer and improve patient outcomes.

In Saudi Arabia, breast cancer ranks as the most common malignancy among women, accounting for a significant proportion of cancer-related morbidity and mortality [10]. The country’s unique demographic and cultural characteristics, coupled with its rapid socioeconomic development, have contributed to distinctive patterns of breast cancer incidence and presentation [11]. Notably, breast cancer patients in Saudi Arabia often present with more advanced disease stages compared to Western populations, underscoring the need for effective early detection and intervention strategies [12,13].

Previous studies documented the epidemiological and clinicopathological features of breast cancer among women in Saudi Arabia [14,15]. Additionally, breast cancer is characterized by its heterogeneity, encompassing a wide range of histological and molecular subtypes with varying prognoses. Many studies have demonstrated that hormone receptor status affects the prognosis, treatment plan, and response of breast cancer [16,17,18], suggesting a more aggressive nature and a worse outcome of the disease in the Saudi population. This diversity necessitates a comprehensive understanding of the biological markers to tailor effective treatment strategies for breast cancer. One such marker, the Ki67 proliferation index, has emerged as a crucial prognostic and predictive tool in breast cancer management [19].

The Ki67 protein is a cellular marker for proliferation, expressed during all active cell cycle phases except the resting phase. High Ki67 levels indicate rapid cell division, which is often associated with more aggressive tumor behavior, such as higher grade and node-positive disease, and poorer clinical outcomes [20]. Therefore, Ki67 has been integrated into breast cancer assessment protocols to help predict disease progression and guide therapeutic decisions [19]. However, its expression and significance can vary across different populations, influenced by genetic, environmental, and lifestyle factors. Therefore, it is crucial to investigate the Ki67 index to interpret its implications accurately and optimize its use in clinical practice.

The primary objective of this study is to thoroughly investigate and analyze the variations in clinical presentations, epidemiological patterns, and pathological characteristics of breast cancer observed within the Saudi population. The secondary objective is to assess the associations between Ki67 levels and various clinicopathological features, including tumor grade, stage, histological type, and molecular subtype among people with breast cancer. We adhered to the STROBE statement (STrengthening the Reporting of OBservational studies in Epidemiology) to ensure this paper meets the standards for reporting observational studies as outlined therein (Supplementary File: Table S1).

## 2. Materials and Methods

### 2.1. Study Design and Setting

This cross-sectional study was conducted between 2016 and 2019 at the Comprehensive Cancer Center of King Fahad Medical City in Riyadh, Kingdom of Saudi Arabia, a central tertiary healthcare facility specializing in oncology. The study enrolled a total of 294 female patients diagnosed with breast cancer at various stages: 12 (4.1%) in Stage I, 79 (26.9%) in Stage II, 79 (26.9%) in Stage III, and 117 (39.8%) in Stage IV. Saudi natives constituted most of the patients (90.1%). Data were collected through a systematic review of medical records and patient assessments, ensuring the inclusion of comprehensive demographic, clinical, and pathological information. The research setting provided access to a diverse patient population, allowing for an in-depth analysis of factors influencing breast cancer outcomes within a specialized oncology care environment.

### 2.2. Data Collection and Registry Protocols

The data for this study were obtained from the cancer registry (tumor board records) of the Comprehensive Cancer Center at King Fahad Medical City. The dataset comprises all patients diagnosed at KFMC during the specified period based on inclusion and exclusion criteria. To ensure the accuracy and consistency of the data, extensive training sessions were conducted for all healthcare providers and administrative personnel involved in the data collection process. These sessions were designed to reinforce adherence to the established protocols and to maintain the integrity of the data.

Upon arriving at the Comprehensive Cancer Center at KFMC, a patient suspected of having breast cancer undergoes a detailed initial consultation. This involves scheduling an appointment, providing a thorough medical history, and undergoing a physical examination by a specialist. The patient then undergoes a series of diagnostic tests, including mammography, ultrasound, and potentially an MRI, to identify the abnormal areas in the breast. If these imaging tests reveal suspicious areas, a biopsy is performed to extract tissue samples for further analysis. The biopsies may include fine-needle aspiration, core needle biopsy, or surgical biopsy. A pathologist then analyzes the collected samples to determine the presence and type of cancer and its grade.

Following the laboratory analysis, additional tests for hormone receptors, Ki67, and HER2 protein are conducted to characterize the cancer further. The cancer records were staged using the American Joint Committee on Cancer (AJCC) TNM (Tumor, Node, Metastasis) Staging System, ensuring all cancer-related data collection components conformed to stringent regulatory and procedural guidelines.

### 2.3. Inclusion and Exclusion Criteria

The inclusion criteria require patients to have thorough documentation of their records, including laboratory and radiological investigations, and to be diagnosed with breast cancer. The available characteristics included age, Body Mass Index (BMI), histology, lymph node involvement, tumor size, site, and extension. We also recorded other related variables such as tumor size, BMI, Estrogen/progesterone receptor (ER/PR) status, HER-2 (human epidermal growth factor receptor 2) status, and pathological TNM staging. We excluded patients who were not diagnosed with breast cancer or had benign histopathology, were not Saudi nationals or residents, were referred to another hospital after initial diagnosis or did not receive treatment at our institution, had incomplete records or missing data on clinical, epidemiological or pathological features, and had a history of previous or concurrent malignancies other than breast cancer.

### 2.4. Operational Definitions and Measurements

Patients included in this study were categorized into four age groups based on their age at diagnosis: ≤40 years, 41–50 years, 51–60 years, and >60 years. Nationality was classified as either Saudi or non-Saudi. BMI was calculated as weight in kilograms divided by height in meters squared (kg/m^2^) and classified into four categories: underweight (BMI < 18.5 kg/m^2^), normal (BMI 18.5–24.9 kg/m^2^), overweight (BMI 25–29.9 kg/m^2^), and obese (BMI ≥ 30 kg/m^2^) [21]. Family history of breast or ovarian cancer was categorized based on the degree of relatives affected: None, 1st Degree (e.g., mother, sister, daughter), 2nd Degree (e.g., grandmother, aunt), 3rd Degree (e.g., cousin, great-grandparent), and Unknown.

Tumor size was classified according to the TNM staging system: T1 (tumor size ≤ 2 cm), T2 (tumor size > 2 cm but ≤5 cm), T3 (tumor size > 5 cm), and T4 (tumor of any size with direct extension to the chest wall and/or skin) [22]. Tumor grade was determined based on histopathological evaluation and categorized as Grade I (well-differentiated/low grade), Grade II (moderately differentiated/intermediate grade), Grade III (poorly differentiated/high grade), and Unknown. Cancer stage was classified using the AJCC staging system as Stage I (early-stage cancer, localized to the breast), Stage II (cancer that has spread to nearby lymph nodes but not to distant parts of the body), Stage III (locally advanced cancer with extensive lymph node involvement), Stage IV (metastatic cancer that has spread to distant organs), and Unknown. The tumor’s location at the time of diagnosis was recorded as Right, Left, or Bilateral.

ER status was categorized as Positive or Negative, as was PR status. HER2 status was also classified as Positive or Negative. The Tumor type was identified based on histological examination and classified into Invasive Ductal Carcinoma (IDC), Invasive Lobular Carcinoma (ILC), Invasive Micropapillary Carcinoma (IMC), and Ductal Carcinoma in situ (DCIS). Molecular subtypes of breast cancer were determined and categorized as Luminal A, Luminal B, HER2-enriched, and Triple-Negative Breast Cancer (TNBC). The primary outcomes of this study centered on the Ki67 proliferation index, a key indicator of tumor cell proliferation. The Ki67 index was categorized into two groups: <20% (indicating lower proliferative activity) and ≥20% (indicating higher proliferative activity) [23].

The <20% vs. ≥20% Ki-67 cutoff is widely used in oncology, particularly in breast cancer, to distinguish low and high proliferative tumors. It holds prognostic and therapeutic significance, with ≥20% linked to aggressive behavior and chemotherapy responsiveness [23]. Efforts to address potential sources of bias include using standardized protocols and statistical adjustments to ensure accurate and reliable results.

### 2.5. Data Analysis

The study utilized both bivariate and multivariate analyses to investigate the characteristics of breast cancer in the Saudi population. Descriptive statistics were used to summarize categorical variables, including frequency and proportion (%). Univariate analyses were conducted to identify factors influencing the Ki67 index, cancer stage, and molecular subtypes. Variables with significant associations in the univariate analyses were included in multivariate logistic regression models to determine independent risk factors associated with elevated Ki67 levels, presenting results as adjusted odds ratios (AOR) with 95% confidence intervals (CI). We considered a comparative analysis between the Ki67 biomarker and different cancer types, where Ki67 is reported in both continuous and categorical forms. A one-way ANOVA test was used to assess the difference in Ki67 levels (as a continuous variable) with breast cancer grades (1 to 3). Additionally, a logistic regression analysis was carried out for Ki67 categorical variables to determine the association between a high Ki67 index with independent factors such as cancer stage, molecular subtypes, tumor grade, and cancer types. Significance was set at a 95% confidence (*p* < 0.05). We used Complete Case Analysis to handle missing data, excluding cases with any missing values. This approach is suitable when missing data are minimal and occur completely at random (MCAR). All statistical analyses were performed using Statistical Packages for Software Sciences (SPSS) version 21 (IBM Corporation, Armonk, NY, USA).

### 2.6. Ethical Approval

The institutional review board (IRB) of King Fahad Medical City (KFMC) approved this study under IRB log number 21-137 (approved on 8 April 2019 and renewed on 28 March 2021). Informed consent was obtained from all individual participants involved in the study. The research was conducted in accordance with the ethical standards set by both the institutional and national research committees, as well as the 1964 Helsinki Declaration and its subsequent amendments or comparable ethical guidelines. Additionally, all procedures adhered to the principles of respect for persons, beneficence, and justice, ensuring the protection of participants’ rights and well-being throughout the study.

## 3. Results

### 3.1. Participants’ Demographic and Clinical Characteristics

This study analyzed 294 patients, with 35.4% of the cohort aged between 51 and 60 (Table 1). Saudi natives constituted most of the patients (90.1%), and over half (67%) were classified as overweight and obese. A family history of breast cancer in first-degree relatives was reported in 7.2% of the cases. Tumor size was predominantly classified as T3 (28.9%), and the cancer grade was mainly Grade III (46.9%). Most patients presented with Stage IV cancer (40%), and the location of cancer was evenly distributed between the left (48.5%) and right (48.5%) breasts. In this study, ER positivity was observed in 63.4% of the patients, PR positivity in 54.3%, and HER2 positivity in 34.9%. A high proliferation index of Ki67 was also detected in 50.7% of the patients. Invasive Ductal Carcinoma was the most prevalent type (83.9%) of breast cancer, and luminal B was identified as the most common molecular subtype cancer (31.0%).

**Figure 1:**
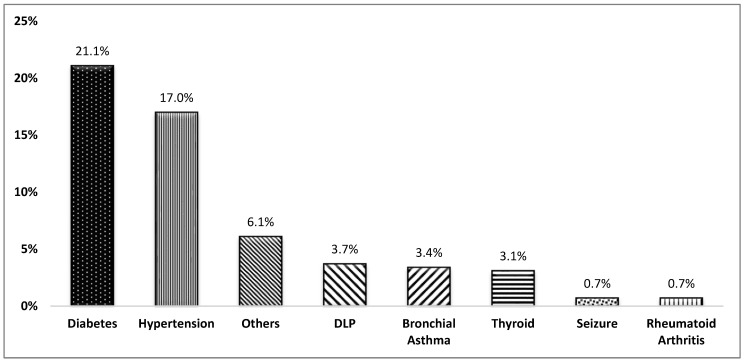
Frequency of co-morbidities among women with breast cancer. DLP: Dyslipidemia.

The most common comorbid conditions among the patients were diabetes (21.1%) and hypertension (17.0%), which are common chronic conditions (Figure 1). Other conditions like dyslipidemia (6.1%) and bronchial asthma (3.4%) were less common but still notable. Seizure disorders and rheumatoid arthritis are rare, each affecting less than 1% of the patients.

### 3.2. Factors Associated with Proliferative Index Ki67 Among Women with Breast Cancer

The Ki67 protein is crucial for breast cancer patients as it indicates the proliferation rate of cancer cells, helping to assess tumor aggressiveness and guide treatment decisions. High Ki67 levels suggest rapid cell division, correlating with more aggressive tumors and a higher risk of recurrence. This study identified a significant relationship between Ki67 expression and tumor grade, with Ki67 levels increasing progressively across grades, reaching the highest levels in Grade III tumors (Table 2). Additionally, significant associations were found between Ki67 expression and tumor stage (*p* = 0.007) and tumor type and molecular subtypes (*p* < 0.001). However, no significant associations were observed between Ki67 expression levels and patient age groups, BMI categories, or tumor size.

**Figure 2:**
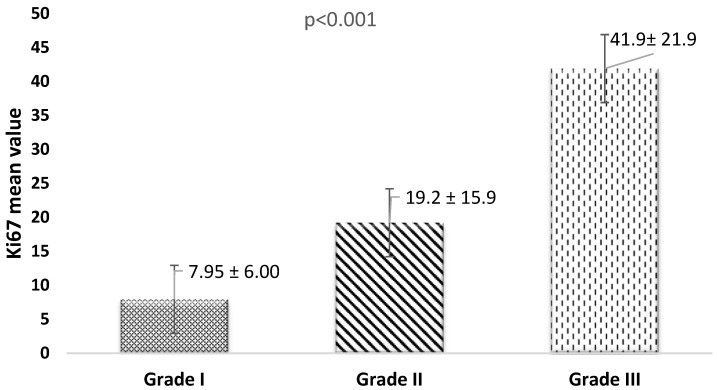
Distribution pattern of the Ki67 index among women with breast cancer grades (1 to 3). *p*-value has been calculated using one-way ANOVA.

Specifically, patients with a Ki67 index of ≥20 were significantly more prevalent among those with Grade III tumors and IDC types. Conversely, a Ki67 index of <20 was more commonly observed in patients with Stage IV cancer. As illustrated in Figure 2, the mean Ki67 value for Grade III tumors was significantly (*p* < 0.001) higher than that for Grade I or Grade II tumors.

### 3.3. Risk of Elevated Ki67 Across Different Tumor Grades, Cancer Stages, and Types

Higher levels of Ki67 are associated with higher grades (Table 3), it was revealed that compared to patients with cancer Grade I, cancer Grade II patients were more likely to have elevated Ki67 by at least 41 times higher (95% CI = 4.36–63.8; *p* = 0.001), while for cancer Grade III patients, likelihood was 7.43 times higher (95% CI =3.68–14.99; *p* < 0.001). Patients with Stage IV cancer were 2.26 times more likely to have an elevated Ki67 than patients with Stage I cancer (95% CI = 1.08–4.73; *p* = 0.03). However, compared to patients with the IDC cancer type, patients with ILC (AOR = 0.07; 95% CI = 0.01–0.63; *p* = 0.01) and other cancer types (AOR = 0.07; 95% CI = 0.01–0.82; *p* = 0.03) were less likely to have elevated Ki67.

### 3.4. Relationship Between Different Cancer Types and Ki67 Biomarker

**Figure 3:**
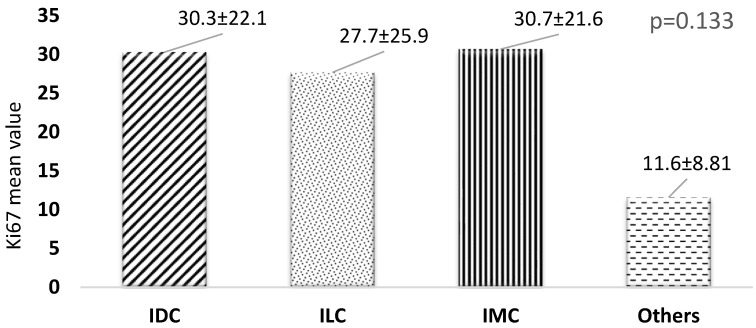
Relationship between different cancer types and Ki67. *p*-value has been calculated using one-way ANOVA. IDC, Invasive Ductal Carcinoma; ILC, Invasive Lobular Carcinoma; IMC, Invasive Micropapillary Carcinoma.

Figure 3 presents a comparative analysis of mean Ki67 values across different cancer types. While there were observable variations in Ki67 expression levels among the cancer types, the differences did not achieve statistical significance (*p* = 0.133). This suggests that Ki67 expression does not vary significantly within the sample analyzed between the cancer types, indicating that other factors may play a more critical role in influencing Ki67 levels.

## 4. Discussion

This study found an association between demographics, tumor characteristics (size, grade, stage), hormonal receptor status (ER, PR, HER2), molecular subtypes, and Ki67 value. The study population presented with a notable trend of advanced stages and higher-grade tumors. The Ki67 index, indicating proliferation rate, was elevated in Luminal B, HER2-enriched, and TNBC. Additionally, Ki67 was valuable for assessing tumor persistence and guiding treatment decisions. While there are consistencies across these studies in linking Ki67 expression with specific tumor characteristics and treatment implications, predictive factors for Ki67 in breast cancer outcomes varied.

An interplay between hormonal, genetic, and metabolic factors contributes to the patho-biological mechanisms underlying our findings [24,25,26]. Hormone receptor-positive tumors, particularly those that are ER- and PR-positive, underscore the significant role of estrogen and progesterone signaling pathways in driving tumor growth [27,28,29,30]. The high Ki67 index found in aggressive subtypes such as HER2-enriched and TNBC reflects increased proliferative activity, often driven by genetic mutations and dysregulation of cell cycle control mechanisms [31]. This aligns with the observed efficacy of hormone therapies in our study, as these pathways are well-documented to be pivotal in hormone receptor-positive breast cancers [32].

The predominance of luminal-B among Saudi patients highlights the need for tailored therapeutic approaches, given its distinct prognosis and treatment response. The notable presence of luminal-A and HER2 subtypes further underscores the importance of molecular profiling in guiding targeted interventions and optimizing patient outcomes. The distribution of molecular subtypes among Saudi patients, with luminal-B being the most common at 31%, luminal-A subtypes at 22.8%, and HER2 at 18.7%. This distribution contrasts with a study from the Riyadh region, where luminal-A was predominant at 58.5%, followed by luminal-B at 14.5%, and HER2 at 12.3% [33]. These findings suggest that a consistent pattern in breast cancer histopathology remains present across different populations in both Saudi Arabia and globally [34,35]. The differences in molecular subtype distributions between our study and the others can be attributed to clinical and statistical factors. Clinically, variations in genetic backgrounds, lifestyle factors, healthcare access, and screening practices can influence subtype prevalence. Statistically, differences in sample size, selection criteria, and analytical methods, such as treating Ki67 as a categorical versus continuous variable, can impact results.

The higher prevalence of HER2-positive tumors in advanced-stage breast cancer suggests a more aggressive disease course, emphasizing the need for early detection and targeted HER2-directed therapies. In this study, we observed a more aggressive tumor biology, with a higher prevalence of HER-2 positivity in Stage 3 and Stage 4 breast cancer. Unlike some studies that reported no significant difference in the prevalence of HER-2 positive tumors between African American and white women [36,37], our findings in the Saudi population indicate a considerable difference. The discrepancy between these studies underscores the necessity for region-specific cancer profiling to optimize therapeutic approaches and improve patient outcomes in different populations.

There may be a greater risk of disease progression and reduced treatment effectiveness in patients with chronic comorbidities. The high prevalence of comorbidities like diabetes and hypertension suggests that chronic metabolic stress and vascular abnormalities may worsen cancer aggressiveness, as shown in other studies [31,32]. The mechanisms behind this association may involve chronic inflammation, insulin resistance, and altered levels of insulin-like growth factors, which can promote tumor growth. These findings underscore the importance of considering chronic disease management while treating patients with breast cancer.

There is a complex relationship between BMI, weight gain, and breast cancer, which varies from population to population. In our study, obesity was prevalent among patients with advanced and high-grade tumors, suggesting that systemic inflammation and altered adipokine levels may create an environment conducive to tumor progression [31,33]. These findings align with previous research that suggests a positive association between obesity and breast cancer in the Korean population [34]. Conversely, other studies have shown that a lower BMI may be linked to an increased risk of breast cancer in African American women [35]. These findings highlight the need to account for regional variations when assessing the influence of BMI on breast cancer risk.

This study highlights the importance of Ki67 for managing advanced breast cancer and understanding tumor progression. Like other studies conducted, the role of Ki67 expression in breast cancer demonstrated varying levels of congruency [36,37]. The study found that tumor grade and stage are robust predictors of elevated Ki67 levels, with advanced stages showing greater significance. Similarly, researchers in two studies found an association between Ki67 and clinicopathological parameters such as age, tumor size, hormone receptor status, and HER2 expression, reporting significant correlations between Ki67 and ER status and HER2 overexpression [29,30]. This suggests that clinical focus on tumor size and grade can be crucial in assessing and managing breast cancer progression [38,39].

Our findings suggest a compelling argument for integrating Ki67 into clinical practice. However, the recent American Joint Committee on Cancer (AJCC) guideline expresses skepticism regarding the technical validity of Ki67 [40]. For instance, the clinical utility of Ki67 in breast cancer care is primarily limited to prognosis assessment in Stage I or II breast cancer. The International Ki67 in Breast Cancer Working Group suggests that further development of automated scoring may help address this limitation [40].

This study may have potential confounders and biases due to a retrospective cross-sectional design, which limits the use of different statistical tests and the ability to establish causality between Ki67 levels and tumor characteristics. For example, confounding variables, such as unaccounted lifestyle factors like alcohol and other substances, may also limit the study’s findings. A selection bias may be present since the data were collected from a single cancer center, potentially affecting the generalizability of findings to the Saudi population. It is also possible that unmeasured confounders, such as genetic predispositions, lifestyle factors, and treatment histories, may also influence the observed associations [40]. Furthermore, recall biases could also exist due to relying on existing medical records, which may contain inconsistencies or missing information. Future prospective studies could improve validity by addressing these limitations.

## 5. Study Limitations and Strengths

This study has several other limitations that should be acknowledged. Firstly, the study did not examine patient care indicators, limiting care quality assessment beyond adherence to treatment guidelines. This gap means that crucial aspects like patient outcomes, satisfaction, and long-term health impacts were not evaluated, potentially missing key dimensions of healthcare quality. The retrospective study design presents inherent challenges, such as potential issues with the documentation and quality of data. One limitation of the study is the presence of missing data. We did not implement methods to address or impute the missing data, which may have affected the robustness and accuracy of our findings. Finally, some important implications of this study will benefit the wide variety of breast cancers affecting patients. Firstly, the varied molecular subtypes in breast cancer patients indicate a need for personalized treatment plans to address the specific characteristics of each patient. The study also found that higher Ki67 levels could be linked with a poorer prognosis, suggesting that Ki67 could be an important factor in guiding treatment decisions and predicting patient outcomes. Therefore, emphasizing Ki67 levels in breast cancer patients will result in a more accurate prognosis and more effective management plans.

## 6. Conclusions

This study underscores the high prevalence of comorbidities such as diabetes, hypertension, and obesity among female breast cancer patients. Correlations were found between the Ki67 proliferation index, tumor grade, and stage, suggesting a link between aggressive tumor behavior and increased levels of Ki67.

Ki67 indices prove to be valuable diagnostic tools that enhance the accuracy of prognosis and facilitate the development of treatment plans tailored to the patient’s individual needs. Healthcare providers can tailor therapeutic strategies to individual patients by incorporating the Ki67 index into diagnostic practices, potentially improving patient outcomes.

## Figures and Tables

**Table 1:** Patient demographics and clinical characteristics (*n* = 294).

Variables	Number (%)
Age Groups	
≤40 years	52 (17.7)
41–50 years	81 (27.6)
51–60 years	104 (35.4)
>60 years Mean, SD [Range]	57 (19.3) 51.58, 11.45 [21, 86]
Nationality	
Saudi	265 (90.1)
Non-Saudi	29 (9.9)
BMI Categories	
Underweight (<18.5 kg/m^2^)	06 (2.0)
Normal (18.5–24.9 kg/m^2^)	59 (20.1)
Overweight (25–29.9 kg/m^2^)	73 (24.8)
Obese (≥30 kg/m^2^) Unknown/missing	124 (42.2) 32 (10.9)
Family History (breast or ovarian cancer)	
None	195 (66.3)
1st Degree	21 (7.2)
2nd Degree	09 (3.1)
3rd Degree	01 (0.3)
Unknown/missing	68 (23.1)
Size of Tumor	
T1	27 (9.2)
T2	71 (24.1)
T3	85 (28.9)
T4	73 (24.8)
Unknown/missing	38 (13.0)
Grade of Tumor	
I	12 (4.1)
II	133 (45.3)
III	138 (46.9)
Unknown/missing	11 (3.7)
Cancer Stages	
Stage I	12 (4.1)
Stage II	79 (26.9)
Stage III	79 (26.9)
Stage IV	117 (40.0)
Unknown/missing	07 (2.1)
Location (at time of diagnosis)	
Right	142 (48.5)
Left	142 (48.5)
Bilateral	10 (3.0)
Estrogen Receptors (ER)	
Positive	184 (63.4)
Negative	110 (36.6)
Progesterone Receptor (PR)	
Positive	159 (54.3)
Negative	135 (45.7)
Human Epidermal Growth Factor Receptor 2 (HER2)	
Positive	106 (34.9)
Negative	188 (65.1)
Ki67 Index	
Low (<20)	90 (30.4)
High (≥20) Unknown/missing	149 (50.7) 58 (19.6)
Tumor Type	
Invasive Ductal Carcinoma	244 (83.9)
Invasive Lobular Carcinoma	21 (7.2)
Invasive Micropapillary Carcinoma	17 (5.8)
Other carcinomas	14 (3.1)
Molecular Subtypes Cancer	
Luminal A	67 (22.8)
Luminal B	91 (31.0)
HER2-enriched	55 (18.7)
TNBC	47 (16.0)
Unknown/missing	34 (11.5)

BMI, Body Mass Index; SD, Standard Deviation; TNBC, Triple-Negative Breast Cancer. We report the following unknown/missing data: BMI categories (10.9%), family history (23.1%), size of tumor (13%), grade (3.7%), stages (2.1%), Ki67 index (19.6%), and molecular subtypes (11.5%).

**Table 2:** Univariate analysis of the factors associated with Ki67 biomarker (*n* = 239).

Factors	Ki67		*p*-Value ^§^
	<20 Unit Number (%) (*n* = 90)	≥20 Unit Number (%) (*n* = 149)	
Age group			
≤40 years	17 (18.9)	25 (16.8)	0.741
41–50 years	22 (24.4)	43 (28.9)	
>50 years	51 (56.7)	81 (54.4)	
BMI Categories			
Non-obese	38 (50.7)	70 (53.4)	0.702
Obese and overweight	37 (49.3)	61 (46.6)	
Size of tumor			
T1	11 (13.1)	10 (8.1)	0.064
T2	30 (35.7)	28 (22.6)	
T3	24 (28.6)	44 (35.5)	
T4	24 (22.6)	42 (33.9)	
Grade of tumor			
I	09 (10.3)	01 (0.7)	<0.001
II	60 (69.0)	49 (33.1)	
III	18 (20.7)	98 (66.2)	
Cancer stage			
Stage I	03 (02.1)	06 (6.7)	0.007
Stage II	46 (31.7)	15 (16.9)	
Stage III	33 (22.8)	33 (37.1)	
Stage IV	63 (43.4)	35 (39.3)	
Cancer type			
IDC	70 (78.7)	127 (86.4)	0.019
ILC	08 (9.0)	09 (6.1)	
IMC	04 (4.5)	10 (6.8)	
Others	07 (7.9)	1 (0.7)	
Molecular subtypes			
Luminal A	67 (77.0)	0	<0.001
Luminal B	10 (11.5)	72 (49.0)	
HER2-Enriched	05 (5.7)	42 (28.6)	
TNBC	05 (5.7)	33 (22.4)	

^§^ *p*-value has been calculated using the chi-square test. BMI, Body Mass Index; DCIS, Ductal Carcinoma in situ; HER2, human epidermal growth factor receptor 2; IDC, Invasive Ductal Carcinoma, ILC; Invasive Lobular Carcinoma; IMC, Invasive Micropapillary Carcinoma; TNBC, Triple-Negative Breast Cancer.

**Table 3:** Multivariate logistic regression analysis to determine the significant independent predictors associated with an elevated Ki67 biomarker (*n* = 236).

Factors	AOR	95% CI	*p*-Value
Tumor Grades			
Grade I	Ref		
Grade II	41.05	4.36–63.73	0.001
Grade III	7.43	3.68–14.99	<0.001
Cancer Stages			
Stage I	Ref		
Stage II	5.10	0.92–28.18	0.06
Stage III	0.49	0.22–1.09	0.08
Stage IV	2.26	1.08–4.73	0.03
Cancer Types			
IDC	Ref		
ILC	0.07	0.01–0.63	0.01
IMC	0.14	0.01–1.53	0.1
Others	0.07	0.01–0.82	0.03
Molecular Subtypes Cancer			
Luminal B	Ref		
HER2-enriched	1.12	0.25–4.96	0.88
TNBC	1.33	0.26–6.59	0.72

Luminal A was excluded from the model as all cases had Ki67 < 20. The model was adjusted for age, Body Mass Index, diabetes, and hypertension. AOR—Adjusted Odds Ratio; CI—Confidence Interval; DCIS, Ductal Carcinoma in situ; HER2, human epidermal growth factor receptor 2; IDC, Invasive Ductal Carcinoma; ILC, Invasive Lobular Carcinoma; IMC, Invasive Micropapillary Carcinoma; TNBC, Triple-Negative Breast Cancer.

## Data Availability

Due to ethical and legal restrictions, the data are not available publicly. The data used and/or analyzed for the current study are available from the corresponding authors upon reasonable request.

## Supplementary Materials

The following supporting information can be downloaded at: https://www.mdpi.com/article/10.3390/healthcare13070737/s1, Table S1: STROBE Statement—Checklist of items included in reports of cross-sectional studies.
